# Ethnic variations in compulsory detention and hospital admission for psychosis across four UK Early Intervention Services

**DOI:** 10.1186/s12888-014-0256-1

**Published:** 2014-09-05

**Authors:** Farhana Mann, Helen L Fisher, Barnaby Major, Jo Lawrence, Andrew Tapfumaneyi, John Joyce, Mark F Hinton, Sonia Johnson, on behalf of the MiData Consortium

**Affiliations:** Division of Psychiatry, UCL, Charles Bell House, 67-73 Riding House Street, London, W1W 7EJ UK; MRC Social, Genetic & Developmental Psychiatry Centre, Institute of Psychiatry, King’s College London, London, UK; East London NHS Foundation Trust, London, UK; South London and Maudsley NHS Foundation Trust, London, UK; Camden and Islington NHS Foundation Trust, London, UK

**Keywords:** Psychosis, Early intervention, Ethnic, Compulsory detention, Admission

## Abstract

**Background:**

Substantial ethnic variations have been found in incidence, pathways to care and outcomes in psychosis. It is unknown whether these remain as marked in the presence of specialist Early Intervention Services (EIS) for psychosis. We present the first UK study exploring ethnic differences in compulsory detention and hospitalization rates for EIS patients. We investigated whether the excess rates of compulsory admission for people from Black groups have persisted following nationwide introduction of EIS. We also explored variations in compulsory admission for other ethnic groups, and differences by gender and diagnosis.

**Methods:**

Four inner-city London EIS teams gathered data from first-presentation psychosis patients between 2004–2009 using the MiData audit tool. Clinical, sociodemographic and pathways to care data were recorded regarding adult patients from eight different ethnic groups at entry to EIS and one year later.

**Results:**

Black African EIS service users had odds of being detained and of being hospitalised three times greater than White British patients, even after adjustment for confounders. This was most marked in Black African women (seven to eight times greater odds than White British women). A post-hoc analysis showed that pathways to care and help-seeking behaviour partially explained these differences.

**Conclusion:**

These findings suggest EIS input in its current form has little impact on higher admission and detention rates in certain Black and minority groups. There is a need to tackle these differences and engage patients earlier, focusing on the needs of men and women from the most persistently affected groups.

## Background

Disparities in the mental health experiences of different ethnic groups have generated much discussion, debate and controversy in recent years. A growing body of research has demonstrated differences in incidence, prognosis and experiences of psychotic illness in Black and minority ethnic (BME) groups compared with the respective native populations [1,2]. A greater risk of adverse pathways to, and contacts with, psychiatric services has also been shown in BME patients [3,4] a source of concern to clinicians, service planners and policy makers, service users and members of the relevant minority groups.

Importantly, significant differences in rates of compulsory detention and hospitalization have been demonstrated in studies of first-episode psychosis (FEP) populations, in the UK and beyond. Though results have shown some variation, the largest and most robust of these studies indicated that Black Caribbean men were 3.5 times more likely to be detained than their White British counterparts [3]. For Black African men, the figure rose to over 4 times as likely. A systematic review conducted by the authors [5] combined existing UK primary studies to give an odds ratio of 2.21 for compulsory detention in BME groups overall compared with White British patients with FEP. With regards to hospital admission, international studies have shown varied results [6-8].

A number of potential explanations have been put forward to account for these differences, ranging from institutional racism in psychiatry to altered illness expression in minority groups, or variation in explanatory models of illness – but most lack corroborating evidence [4,9]. Another possibility is that BME groups may follow more adverse ‘pathways to care’. Mapping pathways to care involves identifying the services involved at any point in the patient’s journey to specialist care, including for example the involvement of general practitioners (GPs), religious organisations or the police. Some evidence has been found that such adverse pathways to care may mediate the relationship between BME status and detention, but that differences are not entirely explained. For instance, the Aetiology and Ethnicity in Schizophrenia and Other Psychoses (AESOP) study conducted regression analyses to adjust for variation in rates of criminal justice referral, help-seeking behaviour, perceived risk to others, diagnosis and employment, but still showed a significantly increased risk of detention in Black African and Caribbean patients [3]. Lawlor’s [10] study (though not exclusive to FEP) suggested help-seeking behaviour and pathways to care may contribute to explaining differences in rates of compulsory admission among women. What has been more consistently demonstrated is a link between adverse contacts and poorer outcomes in psychosis [10,11], highlighting the need to investigate and tackle such inequalities as a priority.

Alongside the drive for equity of mental healthcare across ethnic groups, has been a drive for active early intervention in psychosis [12]. The number of early intervention services (EIS) in operation has grown worldwide since the early 1990s, and English health policy mandated provision of such services between 2004 and 2010. The aim of the EIS model is to intervene early and effectively in young people experiencing their first episode of psychosis. Other than having a specific focus on early psychosis, common desirable features of EI teams include being ‘youth oriented’ (ideally focusing on people aged 14–35) and multidisciplinary, following an assertive outreach model to encourage engagement, and staff members having low caseloads to facilitate intensive provision of a full range of interventions, including psychological and social (typically not more than 15 cases). One of the main aims of EIS has been to engage ‘hard to reach’ patients, including those from BME groups, and to reduce adverse pathways to care and coercive early treatment experiences. Research evidence has demonstrated improvement in outcomes when adopting a specialised early intervention model [13,14], and has found the model to be cost effective [15]. A Cochrane review concluded there is ‘some support’ for early intervention in psychosis but called for better quality research [16]. With regards to ethnic variation in detention/admission in FEP patients, very few studies have been conducted in EIS settings, and our systematic review [5] found no study based on a UK EI population. Thus there is a pressing need to establish whether or not previous ethnic inequities in service use persist despite the EIS approach, which is aimed at engagement and the formation of strong therapeutic relationships and at avoiding admissions, but does not generally incorporate a specific focus on any ethnic group.

Further limitations to the existing evidence on admission and detention rates in the FEP population are methodological weaknesses including small sample sizes and poorly defined, at times meaningless, ethnic categorisations (often merging highly heterogeneous populations) thereby limiting generalisability. In addition, the number of ethnic groups considered has been limited, focusing predominantly on the experiences of Black African and Black Caribbean groups in the UK and Canada, and Maori versus non-Maori in New Zealand. Few studies have taken steps to account for potential confounding or mediating factors, and most have not stratified by gender. This is despite evidence that men and women may have different ethnic disparities in rates of detention [3,17,18].

Our study is the first in the UK to explore ethnic variations in hospital admission and compulsory detention in a FEP population recruited exclusively from EIS teams. For this study, we defined our two main outcome measures as:

1. Whether compulsorily detained in hospital under the UK Mental Health Act by the end of the first year of EIS care. Compulsory admissions taking place during the same treatment episode as recruitment to EIS, but which preceded the EIS taking responsibility for care were included, as an aim for high quality EIS care is engagement as early as possible in the pathway to care.
2. Whether admitted to hospital (voluntarily or compulsorily) by the end of the first year of EIS care.

Our primary hypothesis was that people from black groups (Black British, Black Caribbean and Black African) would be more likely to be admitted to hospital under the Mental Health Act (MHA) than White British patients. As a secondary aim, we explored rates of admission and detention in less well-studied groups including White other, South Asian, Asian other and mixed Black/White patients. We also explored differences between genders in patterns of admission, and whether team structure had any influence on variations in admission rates.

## Method

Multicentre ethical approval was obtained from the Wandsworth Research Ethics Committee, which granted permission for secondary research use of the data for a specific set of research questions, including ethnic variation in admissions and detention. All information, which could lead to patient identification, was removed before the data were made accessible for research purposes. All of the data analysed were collected routinely and used for local service evaluation purposes.

### Setting

We present baseline and 12-month follow-up data gathered from four EIS teams in London, UK, making use of a standardised data collection tool (MiData), which has previously been well described in published papers [19,20]. The teams all aimed to follow the UK Service Implementation Guide [21], delivering a full multidisciplinary service focused on recovery and relapse prevention for two to three years. However, there was relatively little focus on early detection or on detection of at risk mental states. The teams generally accepted patients aged up to 35 within the first three years of psychosis and each covered distinct catchment areas within London. Two different models of EIS delivery are represented. Teams A, B and D operated as stand-alone services, whereas Team C followed a ‘hub and spoke’ model. Specialised, stand-alone services are considered the UK gold standard but are resource-intensive. The ‘hub and spoke’ design featured a centralised hub of EI workers collaborating with dedicated early psychosis care coordinators in the existing community teams (see [22] for further details). ‘Hub and spoke’ designs may require less initial investment of resources, but may find it harder to maintain aspects of high fidelity EI care such as a team approach and multidisciplinary delivery of a full range of interventions tailored to early psychosis. All of the teams served large inner city areas with ethnically diverse populations. Each of the teams represented the standard referral point for all new cases of psychosis entering mental health services in the respective area.

### Sample

The sample comprised adult patients referred to and accepted by the four EIS teams broadly from 2004 to 2009. The aim was to capture all accepted referrals in the time period, but for practical reasons this was not performed uniformly across the teams. Data collection was restricted to certain teams in the earliest stages and in some fledgling services the entire catchment area was not covered initially. However, from 2006 EIS were more broadly implemented and at the very least basic data was collected for all accepted cases. Patients were included at 12-months follow-up if any information was available for them during this period. Exclusion criteria were: aged below 18 years and a diagnosis of learning difficulties or organic (non-functional) psychosis.

### Data collection

The data was entered by clinical and administrative staff onto individual electronic databases, using a standardised computerised assessment package (MiData) and then the anonymised datasets were merged by HLF. MiData is an audit tool developed by the London Early Intervention Network (LEIRN) which comprises a minimum set of assessments used to evaluate first-episode psychosis services (EIS) [20]. All participating staff received training on the package. Data was collected at entry to the EIS (baseline) and one year later. The following measures were utilised in this study:

#### Sociodemographics

Basic sociodemographic information was collected via standardised questions on: gender (male or female); country of birth (born in UK or outside of UK); ethnicity (employing UK Census 2001 categories), marital status (married/cohabiting or single/divorced/separated/widowed); has children (yes or no); current living arrangement (living alone or living with others or roofless/other); education/employment history (ever been in paid employment/had at least high school qualifications [General Certificate of Secondary Education (GCSE) or equivalent] or never been employed/had no qualifications).

For the analyses reported in this paper, only service users from the following self-ascribed ethnic groups were included:

- White British
- White Other (White service users with no parents born in the UK, includes White Irish)
- Mixed Black/White (one parent Black Caribbean or Black African, other parent White)
- South Asian (Bangladeshi, Indian, Pakistani, Sri Lankan origin)
- Other Asian (includes Chinese, Vietnamese, Philippines, Japanese)
- Black British (Black service users born in the UK)
- Black Caribbean (Black and born in the Caribbean)
- Black African (Black and born in Africa)

Those from other ethnic groups were excluded from the study as they belonged to ethnic groups represented in numbers too small to provide sufficient statistical power.

#### Pathways to care

MiData includes a ‘pathways to care’ audit tool which collates information about the different people and services that individuals came into contact with for psychosis prior to entering mental health services (see [20] for full details). This was completed at entry to EIS and included whether the individual saw a General Practitioner or other primary care professional at any point in this pathway (yes or no). Additionally it was noted whether the individual was referred by a criminal justice service (prison, police, or probationary services) to a psychiatrist or mental health service (yes or no) or whether the individual initiated contact themselves (defined as ‘help-seeking’ – yes or no).

#### Admission & detention

A history of any compulsory hospital admissions for psychosis from first contact with mental health services in the current treatment episode for psychosis to the end of the first year of EIS care, was collected from the patients and clinical notes. Whether a patient had been admitted at all in relation to psychosis (i.e. both compulsorily and voluntarily) was also noted, up to and including the first year of EIS care. All detentions in this study were under the civil Sections of the UK Mental Health Act.

#### Clinical diagnosis

ICD-10 [23] diagnosis at 1 year follow-up was extracted from clinical records and confirmed with the EIS consultant psychiatrist. These were then grouped into schizophrenia-spectrum disorders (codes F20-29), affective psychoses (F30.2, F31.2, F31.5, F32.3, F33.3 or F39), and other disorders (all other codes).

### Analysis

All analyses were conducted in Stata version 11.1 for Windows. Descriptive statistics and univariate tests (chi-square/Fisher’s exact/ANOVA) were used to describe and explore any baseline differences in key social and clinical characteristics between ethnic groups. Binary logistic regression was used to explore whether ethnic group was associated with compulsory detention, and then with hospitalization. For the purposes of the analysis, each team was compared with Team A (largest team). All ethnic groups were compared with the White British group in each model. The analysis was conducted for men and women separately. Adjustments were made for gender (only in the models for the full sample), age at referral to EIS, team and diagnosis, as these may influence detention rates [3,18,24].

As there was a large quantity of missing data for the variables concerning criminal justice referral, GP involvement, and help-seeking behaviour in two of the teams, a subgroup analysis was conducted just on the two remaining teams combined (Team A and Team D) in order to investigate whether these variables may account for any relationship found between ethnicity and sectioning/admission. These factors have been demonstrated as potentially accounting for some of the variation between ethnic groups in rates of being detained in previous studies [3,25,26]. Logistic regression analysis was conducted on the subgroup to explore if ethnic differences in rates persisted when adjustments were made for pathway to care and help-seeking behaviour.

### Missing data

In keeping with the original paper describing the MiData tool [20], some variables suffered from poor completion rates. This is despite the tool being relatively user-friendly and focused on clinically important parameters. For the main outcome variables of detention and admission by 12 months follow-up, comparisons were made between those patients with missing data (n = 44 for detention outcome and n = 19 for admission outcome) and those with data available (n = 630 for detention outcome and n = 655 for admission outcome) for basic social and clinical factors. There were no statistically significant differences with regard to age, gender, marital status, employment or education, diagnosis or ethnicity when the data was missing versus not missing for detention or admission (all p values >0.05). Rates of missing data per variable are listed in Table 1.

**Table 1:** **Completeness of relevant MiData variables**

**Item**	**Completion rate (N = 674) n (%)**
Ethnicity	674 (100)
Age at referral	672 (99.7)
Gender	672 (99.7)
Diagnosis	663 (98.3)
Ever admitted?	655 (97.2)
Ever sectioned?	630 (93.5)
Ever employed or educated to GCSEs?	617 (91.5)
Criminal justice referral?	594 (88.1)
Marital status	552 (85.7)
Duration of untreated psychosis	527 (78.1)
GP in pathway?	528 (78.3)
Living arrangement	511 (75.8)
Self referral?	502 (74.5)

GCSE, General Certificate of Secondary Education.

## Results

### Sample characteristics

Over the study period, 714 patients meeting the initial inclusion criteria presented to the four services. Forty cases were removed from the sample at this stage, as they belonged to ethnic groups represented in only very small numbers. These groups were Other, Mixed other, Black other and Mixed White and Asian. This gave a final sample size of 674 patients across 8 ethnic groups. The sample comprised 438 (65.0%) men and the mean age at referral was 24 years (S.D. 4.5, range 18–35). Just over a fifth of patients lived on their own (21.8%), and the majority had been employed at some point in their lives or were educated up to at least GCSE level (94.1%).

### Sociodemographic and clinical characteristics by ethnic group

The basic demographic and clinical characteristics of each ethnic group are presented in Table 2. The largest ethnic group in the sample overall was Black African (27.9%), followed by White British (23.4%). The smallest group represented was the Asian other group (4.3%) which included people who described themselves as Chinese, Vietnamese and Filipino. Comparisons across ethnic groups for basic social characteristics showed no significant differences at p = 0.05 (Table 2).

**Table 2:** **Sociodemographic and clinical characteristics of the people with psychosis across the eight ethnic groups**

**Demographic/clinical variable**	**White British**	**White other**	**Mixed Black/White**	**South Asian**	**Asian other**	**Black British**	**Black Caribbean**	**Black African**	**Test statistic** ^**1**^	**p**
	N = 158	N =93	N =36	N =37	N =29	N =55	N =78	N =188		
Age at referral: M (S.D.)	23.6 (4.2)	23.6 (4.6)	22.9 (4.2)	24.6 (4.5)	25.9 (5.5)	23.5 (4.5)	24.4 (4.7)	24.2 (4.6)	4.7	0.702
Gender n (%)										
Male	114 (72.6)	58 (62.4)	20 (55.6)	25 (29.4)	19 (65.5)	36 (65.5)	53 (68.0)	113 (60.1)	8.3	0.307
Marital Status n (%)										
Married	10 (7.9)	13 (14.9)	1 (3.2)	5 (14.7)	5 (20.8)	1 (3.1)	6 (9.5)	12 (7.8)	11.4	0.123
Ever employed or achieved GCSEs? n (%)										
Yes	137 (94.5)	85 (95.5)	32 (94.1)	35 (97.2)	25 (92.6)	44 (88.0)	66 (95.7)	154 (92.2)	4.9	0.678
Living Arrangement n (%)										
Living alone	18 (15.5)	16 (18.6)	5 (19.3)	5 (16.1)	3 (13.6)	10 (30.3)	16 (27.6)	37 (26.6)	19.2	0.157
Living with others	83 (71.5)	63 (73.2)	20 (76.9)	25 (80.7)	19 (86.4)	19 (57.6)	38 (65.5)	95 (68.4)		
Roofless/Other	15 (12.9)	7 (8.1)	1 (3.9)	1 (3.2)	0 (0)	4 (12.1)	4 (6.9)	7 (5.0)		
Diagnosis n (%)										
Schizophrenia-spectrum	114 (73.6)	63 (69.2)	28 (77.8)	30 (83.3)	20 (69.0)	41 (74.6)	55 (72.4)	149 (80.5)	27.8	0.015
Affective psychosis	18 (11.6)	25 (27.5)	5 (13.9)	5 (13.9)	5 (17.2)	7 (12.7)	11 (14.5)	25 (13.5)		
Other	23 (14.8)	3 (3.3)	3 (8.3)	1 (2.8)	4 (13.8)	7 (12.7)	10 (13.2)	11 (6.0)		
GP in pathway? n (%)										
Yes	61 (50.0)	36 (42.3)	14 (48.3)	18 (56.3)	7 (29.7)	9 (28.1)	23 (37.7)	49 (34.3)	14.4	0.045
Self-referral? n (%)										
Yes	46 (40.0)	39 (46.4)	5 (17.9)	13 (41.9)	6 (26.1)	13 (41.9)	20 (34.5)	41 (31.1)	12.0	0.099
Criminal Justice Referral? n (%)										
Yes	11 (8.2)	13 (14.3)	4 (13.3)	3 (11.5)	3 (6.0)	11 (15.9)	40 (25.0)	89 (15.0)	21.3	0.003
Compulsory detention? n (%)										
Yes	53 (34.9)	32 (36.8)	14 (41.2)	15 (44.1)	10 (37.0)	27 (52.9)	32 (46.4)	105 (59.7)	27.2	<0.001
Admitted? n (%)										
Yes	84 (53.9)	57 (63.3)	20 (57.1)	20 (55.6)	17 (58.6)	41 (77.4)	44 (60.3)	143 (78.1)	33.6	<0.001

M, mean. SD standard deviation. GCSE General Certificate of Secondary Education. GP, general practitioner.

^1^The p-values are derived from significance tests for the association between each variable and ethnic group, using ANOVA for age at referral and χ^2^ /Fisher’s exact for the rest of the variables.

Rates of schizophrenia-spectrum diagnoses were highest amongst the South Asian (83.3%) and Black African (80.5%) groups, and lowest in those classified as White other (69.2%). This latter group also had the highest rate of affective psychosis. With regards to GP involvement in the pathway to care, the lowest rates were observed amongst Black British, Black Caribbean and Black African cases – the highest in the South Asian group. In addition, police involvement and criminal justice referral were highest in the Black African (30.1%) and Caribbean (25.0%) groups respectively. There were no significant differences between ethnic groups with regards to rates of self-referral.

### Compulsory detention

In total, by 12 months follow-up, 288 service users had been compulsorily detained under the UK Mental Health Act for psychosis (42.7%). The group showing the highest rate of detention overall was the Black African group (59.7%), followed by the Black British group (52.9%). The lowest rates of detention were in the White British (34.9%) and White other groups (53.9%). Rates of detention per ethnic group are included in Table 2. Table 3 shows the associations between ethnic group and detention for men and women.

**Table 3:** **Adjusted odds ratios for compulsory detention by ethnicity, team and diagnosis**

	**Male**			**Female**		
	**Adjusted OR**	**95 % CI**	**p value**	**Adjusted OR**	**95 % CI**	**p value**
White other vs. White British	0.71	0.35-1.43	0.335	3.33	1.13-9.77	0.028
Mixed Black/White vs. White British	0.68	0.24-1.97	0.481	6.71	1.73-25.9	0.006
South Asian vs. White British	1.56	0.61-4.02	0.353	2.28	0.52-10.0	0.276
Asian other vs. White British	1.13	0.39-3.23	0.819	1.81	0.34-9.46	0.484
Black British vs. White British	1.76	0.78-3.98	0.176	3.78	1.14-12.5	0.030
Black Caribbean vs. White British	1.24	0.61-2.53	0.546	3.93	1.21-12.7	0.022
Black African vs. White British	2.13	1.22-3.71	0.007	7.25	2.86-18.4	0.000
Age at referral	1.01	0.96-1.05	0.795	0.99	0.93-1.06	0.735
Team B vs. Team A	0.65	0.34-1.25	0.200	0.44	0.17-1.09	0.075
Team C (hub and spoke) vs. Team A	1.14	0.70-1.85	0.601	1.40	0.66-2.95	0.375
Team D vs. Team A	0.62	0.30-1.26	0.183	0.50	0.19-1.34	0.169
Affective vs. schizophrenia-spectrum	0.86	0.44-1.70	0.665	0.86	0.44-1.70	0.665
Other vs. schizophrenia-spectrum	0.78	0.39-1.57	0.486	0.78	0.39-1.57	0.486

CI, confidence interval. OR, odds ratio. All OR adjusted for the other variables listed in the table.

Compared with the reference group, White British service users, the most highly significant difference in compulsory detention rates was for the Black African group, in whom the overall odds of admission were nearly three times as high. Black African men had odds of admission more than twice that for White British men, whilst for Black African women the figure rose to over seven times compared with White British women with both differences being highly significant. For men in the Black British group, the difference did not reach statistical significance, but for Black British and Black Caribbean women the odds of involuntary admission were close to four times greater than for White British women. The figure for Black Caribbean males again did not reach statistical significance. The odds of compulsory admission were over six times greater for mixed Black/White women than for White British women.

Similarly, within the White Other group, women were found to have over three times increased odds of compulsory admission compared with White British women, though the men showed slightly lower odds than their White British counterparts, which did not reach statistical significance. Asian groups did not show significantly different odds of detention. The hub and spoke team showed the highest odds of compulsory detention but this did not reach statistical significance (OR 1.21 compared to Team 1). The odds of being detained did not differ with age or diagnosis.

### Hospital admission

Overall, 426 (63.2%) patients were admitted to hospital for psychosis by 12 months follow-up. Table 4 presents the adjusted odds ratios in men and women.

**Table 4:** **Adjusted odds ratios for hospital admission by ethnicity, team and diagnosis**

	**Male**			**Female**		
	**Adjusted OR**	**95% CI**	**p value**	**Adjusted OR**	**95% CI**	**p value**
White other vs. White British	0.93	0.47-1.84	0.824	4.93	1.63-14.89	0.005
Mixed Black/White vs. White British	1.05	0.37-2.94	0.927	1.64	0.48-5.68	0.433
South Asian vs. White British	1.16	0.45-2.99	0.752	0.60	0.13-2.78	0.511
Asian other vs. White British	1.94	0.64-5.86	0.240	1.07	0.25-4.53	0.928
Black British vs. White British	1.99	0.83-4.77	0.122	10.05	1.95-51.90	0.006
Black Caribbean vs. White British	0.79	0.39-1.59	0.506	2.19	0.74-6.48	0.157
Black African vs. White British	2.03	1.14-3.63	0.017	8.43	3.25-21.83	<0.001
Age at referral	1.01	0.96-1.06	0.705	1.00	0.93-1.08	0.908
Team B vs. Team A	1.33	0.72-2.47	0.362	0.77	0.31-1.89	0.562
Team C (hub and spoke) vs. Team A	1.49	0.89-2.48	0.127	1.29	0.57-2.94	0.542
Team D vs. Team A	0.53	0.26-1.08	0.082	0.60	0.20-1.74	0.342
Affective vs. schizophrenia-spectrum	0.84	0.41-1.74	0.646	0.85	0.41-1.76	0.655
Other vs. schizophrenia-spectrum	0.86	0.42-1.75	0.676	0.60	0.18-1.97	0.404

CI, confidence interval. OR, odds ratio. All OR adjusted for the other variables listed in the table.

The regression models for hospital admission produced fairly similar results to those for compulsory detention, with a few exceptions. Women from the White other group had odds of admission that were over four times higher than their White British counterparts, which was in contrast to the men in this group (OR 0.93, non-significant). As with detention rates, the Black African group showed significantly increased admission risk, for both men and women. The most marked differences were for Black British women and Black African women who had over ten and eight times greater odds of admission than White British women, respectively. There was no significant association between odds of admission and age or diagnosis. Compared with Team A, patients in the hub and spoke team (Team C) showed 1.5 times increased odds of admission in men, and 1.3 times in women, though this did not reach statistical significance.

### Compulsory detention and hospitalisation in the subgroup: GP involvement, criminal justice and self-referral

A subgroup analysis was carried out with Teams A and D combined: these were the team for which pathway to care data was relatively complete. There were not however sufficient numbers to permit separate analysis by gender. Criminal justice referral was associated with much increased odds of detention in this sample (OR 17.2, 95% CI 6.0-49.5). In contrast, self-referral (OR 0.23 95% CI 0.13-0.39) and GP involvement in the pathway (OR 0.30, 95% CI 0.18-0.50) were each linked with a reduced risk of detention. All of these associations were highly significant (p < 0.0005). Associations between ethnic group and both hospitalisation and detention are presented in Table 5 with adjustment for demographic factors and GP involvement, self-referral and criminal justice referral.

**Table 5:** **Logistic regression models of factors associated with detention and admission, all ethnicities compared with the White British psychosis patients**

**Ethnic group**	**Detention n (%)**	**Adjusted OR (95 % CI)**	**p**	**Admitted n (%)**	**Adjusted OR (95% CI)**	**p**
Step 1. Adjusted for age, gender, diagnosis						
White British	20 (26.0)	Reference	-	33 (42.9)	Reference	-
White other	20 (37.7)	1.8 (0.8-4.0)	0.127	30 (56.6)	1.8 (0.9-3.7)	0.117
Mixed Black/White	6 (54.6)	3.4 (0.9-12.6)	0.065	6 (54.6)	1.4 (0.4-5.2)	0.573
South Asian	9 (45.0)	2.5 (0.9-7.0)	0.086	9 (45.0)	1.1 (0.4-3.0)	0.878
Asian other	5 (35.7)	1.6 (0.5-5.3)	0.472	8 (57.1)	1.8 (0.6-5.8)	0.329
Black British	5 (41.7)	2.0 (0.6-7.2)	0.278	7 (58.3)	1.7 (0.5-5.7)	0.428
Black Caribbean	9 (40.9)	2.0 (0.7-5.5)	0.174	12 (54.6)	1.6 (0.6-4.3)	0.329
Black African	58 (63.7)	5.4 (2.7-10.7)	<0.001	72 (79.1)	4.9 (2.4-9.7)	<0.001
Step 2. Adjusted for age, gender, diagnosis plus GP involvement, criminal justice referral and help-seeking						
White British	20 (26.0)	Reference	**-**	33 (42.9)	Reference	**-**
White other	20 (37.7)	1.1 (0.4-2.6)	0.908	30 (56.6)	1.3 (0.5-3.2)	0.534
Mixed Black/White	6 (54.6)	2.2 (0.5-10.3)	0.297	6 (54.6)	0.7 (0.2-3.4)	0.697
South Asian	9 (45.0)	2.5 (0.8-7.9)	0.108	9 (45.0)	1.1 (0.3-3.5)	0.870
Asian other	5 (35.7)	1.1 (0.3-3.9)	0.938	8 (57.1)	1.1 (0.3-3.9)	0.935
Black British	5 (41.7)	1.7 (0.4-7.2)	0.467	7 (58.3)	1.6 (0.4-7.0)	0.513
Black Caribbean	9 (40.9)	1.9 (0.5-6.6)	0.315	12 (54.6)	1.6 (0.4-5.8)	0.507
Black African	58 (63.7)	2.8 (1.3-6.4)	0.012	72 (79.1)	3.1 (1.3-3.1)	0.009

Note. Teams A and D combined, n = 302. Ethnicity by gender interaction not significant for any ethnic groups.

CI, confidence interval. OR, odds ratio. All OR adjusted for age at referral to EIS and also for ethnicity, team and diagnosis, where this wasn’t the variable of interest for the comparison.

As the ethnic makeup of this subsample was slightly different from the original group, the odds of detention for each ethnic group were calculated again compared with White British service users. After adjusting for basic demographics, gender and diagnosis, the odds of being detained for the Black African group were more than five times those for the White British group (p < 0.0005). The mixed Black/White group showed significantly increased odds of detention at over four times the White British group. The other ethnic groups all showed a trend towards increased detention, but none were statistically significant.

With the addition of the GP, criminal justice and self-referral variables, the mixed race group no longer showed significantly greater odds of detention. The Black African group showed a reduction in odds of detention to less than three times as likely as White British patients, which remained a significant difference (p = 0.013) after adjusting for these three additional factors (Table 5).

Unadjusted, only the Black African group showed significantly greater odds of hospital admission (voluntary or compulsory) compared to White British patients (over five times). Adjusting for basic demographics, diagnosis and the three pathway/help-seeking variables showed a similar result to that for detention (Table 5). The odds of admission in Black African patients reduced to just over three times that of the White British group but remained significant (p = 0.009).

## Discussion

This study moves beyond previous research in a number of important respects. Firstly, it is the largest UK study comparing detention/admission rates in FEP patients. It is also the first such UK study to be conducted across Early Intervention Services. Furthermore, we took steps to classify ethnicity into more meaningful categories, including groups beyond crude ‘black’ and ‘white’ classifications, and where possible presented results stratified by gender. In keeping with our original hypothesis, we found Black African patients significantly more likely to be detained in this FEP sample compared to their White British counterparts, though this did not extend to Black British and Caribbean groups. The most markedly increased rates were observed in Black African women, in whom the odds of detention were more than seven times higher than White British women, up to a year into EIS care.

### Different team structures

Each included team operated using a slightly different model and was from a geographically distinct region of London. There were differences with regard to ethnic mix between groups, which reflected the local populations served by each team. The only team showing increased odds of admission to hospital was Team C (1.5 times that of Team A for men and women combined, p = 0.067), which was also the only ‘hub and spoke’ team. This increased admission rate may be explained by the lack of a specialised dedicated team to deliver assertive input for FEP patients at the earliest stages. Overall, though the different approaches had modest impacts on admission and detention rates; more focused comparisons of each could be a direction for future work.

### Compulsory detention

There were marked differences in detention rates by ethnic group, even after adjustments for social and clinical characteristics. Overall, the results share some similarities with the pre-EIS AESOP [3] study findings (Black African and Caribbean FEP detained more than White British patients). The AESOP study was in fact partially based in an overlapping region of London, but was conducted prior to the introduction of EIS teams. Of note, the odds of detention appeared higher in Black Caribbean women compared to men in our study (adjusted OR 3.9 and 1.2 respectively). This is in reverse to the corresponding figures in the AESOP study (OR 1.3 and 3.5) [3]. Our findings also challenge earlier work that suggested such ethnic differences only appeared after years of contact with psychiatric services [7,26,27]. The most notable result was in the Black African group, who were together nearly three times more likely to be compulsorily detained compared with their White British counterparts. However, of note (and in contrast to the AESOP study), the odds were strikingly highest in Black African women, who had over seven times greater odds of being detained. Interestingly, this difference appeared to extend to mixed Black/White women (nearly seven times increased odds) and Black Caribbean and Black British women also showed nearly four times higher odds of being detained compared to White British females. Previous work has not looked specifically at the experiences of mixed race individuals. Furthermore, by including patients from the White Other category, we showed women in this group also had significantly raised odds of involuntary admission (over three times White British rates). The observation that disparities between Black Africans and White British patients do *not* appear to have reduced, and in women may appear *greater*, after the establishment of EIS teams is of concern and suggests more needs to be done to address the needs of BME groups within these services.

Few studies have explored the needs of women with FEP specifically, but a recent study of women in acute mental health crises highlighted increased odds of detention in BME women, particularly Black Africans [25]. We demonstrate that this is highly relevant for young women at the earliest stages of psychotic illness. Much recent attention has focused on the adverse routes of contact experienced by Black Caribbean men (with good reason) but in light of these findings, the needs of women in a number of ethnic groups also need to be highlighted and tackled. A previous Dutch study showed significantly increased rates of detention in young Surinamese women, but not Surinamese men [18]. The authors of this study pointed out similarities between migrant groups in the Netherlands and Black Caribbeans in the UK. Interestingly, in our study Black Caribbean men did *not* show significantly increased odds of detention though Black African men did. The former is in contrast to findings from AESOP, and one could speculate high profile cases and increased training/awareness of the needs of young men from this group may be starting to have an impact. In addition, the Black Caribbean population may itself be changing, in terms of willingness to seek help/attitudes to services and perhaps becoming more integrated. Indeed, Black British men show similar odds of detention to those in the Black Caribbean group. Early Intervention Service staff may be less aware of the high rates of adverse contacts amongst Black African and mixed race women that we have observed here.

The subgroup analysis that was conducted went some way towards identifying a possible explanation for the increased rates of detention in Black African patients. This group showed lower rates of involvement of GPs in their pathways to EIS (in keeping with previous research) as well as significantly less self-referral for help, and more criminal justice referrals. However, this only partially explained the differences, with this ethnic group still nearly three times more likely to be detained than White British patients. A systematic review of pathways to care for FEP patients could not find consistent results for ethnic/gender/socioeconomic determinants [24] but Lawlor et al’s study [25] of women from ethnic minorities experiencing a mental health crisis showed help-seeking behaviour to be an explanatory factor in variation in rates of compulsory detention. Beyond this, knowledge of what drives help-seeking behaviour itself needs to be explored and addressed. Variation in explanatory models of psychosis, for example, is a possible target for public education (31).

The needs of the non-British White population have received relatively little attention in the literature, partly because they include large groups that have migrated to the UK relatively recently. One UK study [25] showed higher rates of detention in this group (non-FEP women). The group in this study is very heterogeneous, including Turkish, Polish and Irish patients. However, the finding that women in this group showed significantly raised rates of involuntary admission highlights the need to conduct more research to better understand the needs of these communities.

### Hospital admission

Risk of hospitalisation has been less studied than detention, but admission to hospital is generally considered a less desirable outcome than community management. Of note, our population showed a high rate of admission (63%) despite the presence of EIS. This suggests a need to review what could be done to bring these levels down, as beginning EIS contact with an acute admission to hospital is likely to be a traumatic experience for service users and their families. A systematic review by the authors [5] revealed only three existing papers comparing rates of admission in FEP by ethnicity, which showed mixed results and had several methodological weaknesses. Our larger, more robust study revealed significantly increased odds of admission in FEP in the Black African group overall (adjusted OR 3.2) within an EIS setting. For women, the odds of admission were over eight times higher for Black Africans than White British women. For men, the figure was twice as high as their White British counterparts. Black British women also showed markedly increased odds of admission (over ten times), though the increased rate did not reach significance for Black British men. In keeping with the detention findings, women from the White other group were more than four times as likely as White British women to be admitted to hospital. Of note, the Black Caribbean group (men and women) did not show significantly increased admission rates. It should be noted the relevant confidence intervals are fairly wide, with the possibility that sample sizes may not have been large enough to capture real differences in some groups. Such variation again highlights the importance of not grouping together all Black or all White service users, as vital information about particular groups’ experiences may be lost. As with detention, increased criminal justice involvement partially explained the increased rates seen, along with reduced GP involvement, and less self-referral (subgroup analysis).

### Explaining the differences

A number of explanations have been proposed for increased admission/detention in ethnic minorities, with racism and racial stereotyping of Black and minority ethnic patients one of the most frequently proposed explanations, according to a systematic review by Singh et al. [4]. However, there is limited primary evidence to support such views (though this does not rule out a role for racism/perceived racism) and the reality is likely to be more complex. Other explanations have included higher rates of psychosis, different clinical presentations in BME patients and different explanatory models of illness [7,26,28]. In addition, increasing dissatisfaction with and suspicion of services over time has been suggested as a reason that the differences between BME and White British patients increase over time [26,29].

The reasons why, for example, South Asian women with FEP are more likely to involve a GP in their care, or more likely to self-refer compared with Black African women are likely to be complex and multifactorial [30]. This paper highlights the marked variation between ethnic minorities and the need for more specific, focused research (including qualitative work) into the different experiences of better defined ethnic groups (including mixed race), with differentiation by gender.

### Implications for Early Intervention Services (EIS)

In an increasingly outcome-driven and evidence-based era, EIS need to demonstrate a significant positive impact on detecting and treating psychosis early, across all groups. Our findings, when compared with UK studies from the pre-EIS era [5], suggest no improvement in the inequality between Black African patients with FEP and White British patients in terms of experiences of admission and detention. The high rates of detention and hospital admission overall are likely to have substantial implications for continuing engagement. The rate of detention is particularly elevated in Black African patients at 60% (Table 2). A disconcerting finding is of even higher rates in certain groups than prior to introduction of EIS, especially in women. While there is overall evidence that the EIS model is a cost-effective [31] means of engaging hard-to-reach young people, it would seem not all groups are being reached in ways that minimise stigma and trauma. Of note, a recent systematic review of initiatives to shorten DUP [32] concluded that establishing dedicated services for people with FEP does not in itself reduce DUP. This is despite evidence that longer DUP is associated with poorer outcomes [33,34].

An important issue is that most EI services in England are primarily recovery-focused; they pick up young people with psychosis after they have had contact with mental health services and are not resourced to actively seek people at high risk or in the early stages of psychosis before help is sought from health services. In addition, they do not generally operate with an ethnic focus. There is now a need to consider how early engagement with services by community routes might be promoted, especially for those groups who currently tend to reach services via coercive pathways. People from some minority groups or their families may for example choose to approach trusted religious or other community figures and organisations rather than health professionals when experiencing psychotic symptoms. Seeking to open up links with such figures/establishments and to develop a mutual understanding of the help needed might result in EIS services appearing more relevant and accessible to the communities they serve. It appears imperative for EIS to focus more closely on the ethnic groups that show the most persistent and marked differences in adverse routes.

### Further clinical, research and policy implications

In Britain, the former government’s five year ‘Delivering Race Equality’ (DRE) programme stated that one of its specific aims was reducing the ‘disproportionate’ rates of compulsory detention of BME patients in inpatient units [11]. However, the fifth annual ‘Count Me In’ census (2010) showed no such shift despite a number of community and hospital-based interventions across the country. Conclusions in a review of the DRE’s impact included identifying a need for better quality research into these differences and the importance of considering individual BME groups distinctly from one another. Our study findings support this drive for more specific, well-designed research into the needs of ethnic minorities with FEP attending EIS. It will be important to better understand the reasons certain groups do *not* experience as much adverse contact as others, as well as the excess of adverse experiences in others. Furthermore, given that some of our findings are in contrast to those from the AESOP study, attempts to replicate results will be helpful.

The markedly raised rate of detention and admission in certain female groups, over and above men, was an unexpected but important finding. It demonstrates adverse contact routes are not just a problem affecting ‘angry young men’, and the factors underlying these differences need to be further explored, in order to be tackled. Adapting psychological approaches to patients, taking into account their ethnic origin, has been demonstrated to improve outcomes [35] in psychosis. This has not been investigated in an EIS setting. As discussed earlier, EI services do not currently operate with an ethnic focus. Addressing this should involve consulting directly with service users, carers and other community members regarding their views and experiences of early intervention. In addition, cultural differences in how women with mental illness are perceived in their communities may be an important area of future study. A recent analysis of UK data on use of the Mental Health Act (4423 assessments over four years) found female gender to be a strong predictor of detention [36]. It has been argued that women with mental illness who are also from ethnic minorities suffer a so-called ‘triple disadvantage’ when it comes to engaging with services [37]. One of the key features of the EIS model is following an ‘assertive outreach’ approach. Not adhering to this, for example as a result of excessively large case loads, could be hypothesized to particularly affect the engagement of ethnic minority patients. These differences could be explored with a combination of quantitative and qualitative research. The latter may provide clues to the potential mechanisms underlying ethnic inequalities, including among previously under-investigated groups such as Turkish, Polish or Eastern European patients. A recent series of interviews with young people with FEP highlighted the importance of engaging families and promoting better communications with key workers [38]. Future work could look into the way EIS engage women, in particular those from ethnic minorities, as well as further examine attitudes to help-seeking in different minority groups. This would equip us better to develop early detection initiatives targeting specific communities.

#### Limitations

One of the most notable limitations is the high levels of missing data for certain variables. As a result we were unable to explore differences in duration of untreated psychosis or other potential risk factors (violence, suicidal ideation, severity of symptoms). Our study has highlighted some of the difficulties inherent in relying on busy clinicians to collect routine data to be used in research. The MiData package is a useful tool enabling wide-scale research but it has proved difficult to attain satisfactory completion rates for some assessments. More dedicated support staff or even streamlining the database further may enhance future data capture.

In addition, despite our efforts to employ more meaningful ethnic categories, some of the groups were fairly heterogeneous including the largest Black African group, which included patients from a number of different African countries, with different cultures, religions and geographical locations. The White other group also consisted of patients from cultures and countries as far apart as Iran and Poland. Also, owing to the small number of patients with affective psychosis in some ethnic groups, the categories of manic and depressive psychosis were merged.

Our post-hoc subgroup analysis could not be stratified by gender owing to the smaller sample size, and the exclusion of two teams reduces its generalisability. There were also no adjustments made for multiple testing and thus some associations may have been over-estimated. With regard to generalisability of the study as a whole, the four teams studied were from urban parts of London with a relatively dense ethnic minority mix and are not typical of the UK as a whole suggesting future research needs to be conducted in more disparate and rural locations around the UK.

Finally, an issue affecting most existing studies in this area is that of the denominator, as this should ideally be the population actually assessed under the MHA, not just those detained. Unfortunately no such data are available. It has previously been proposed that data relating to both assessment and detention should be routinely and centrally collated [4] to facilitate more accurate comparisons.

## Conclusion

This study shows marked differences in rates of detention and admission in certain ethnic minorities (notably Black African patients) compared with White British patients with FEP, across four EIS teams. Adverse contacts were also found for other less well-studied groups including women classed as White other or mixed race. The high rates were only partially explained by criminal justice referral, reduced self-referral and less GP involvement in these groups. There is a need for more detailed hypothesis-driven research to better understand these disparities as a basis for improving services and promoting equality of care regardless of gender or ethnic categorisation.
